# Derangements, Primary and Reflex, of the Organs of Digestion

**Published:** 1842-01-01

**Authors:** 


### Derangements, Primary and Reflex, of the Organs of Digestion. By *Robert Dick*, M.D. 8vo. Edinb. 1840.

We have perused this work with pleasure and instruction. It is decidedly the best compilation in the English language on the extensive class of disorders and diseases comprehended under the term dyspepsia, united with a very large proportion of original matter, both in the form of able comments on other writers, and practical information derived from the author's own experience. But the mass of compiled and original matters is so well mixed and amalgamated as to defy the powers of the most laborious German analyst to separate the one from the other. The *former* we cannot introduce without the latter, and *both* we are unable to review. The work itself, indeed, is a very comprehensive review, critical, analytical, synthetical, and auto-graphical.

We shall introduce two or three cases, or passages, from the author's own note-book, that may not be without interest.

Case 1. *Nitrate of Silver.*—A young gentleman, who had stomach ailments almost from infancy. The most distressing symptom, however, was eructation of an acid and burning fluid and gas from the stomach, which often commenced even during meals, and continued for hours afterwards. Yet his bowels were regular, urine natural, and the functions of the skin normal, with sound sleep—and no apparent disorder or disease of any other organ in the body.

"The case of this young gentleman appearing to me to betray an union of morbid secretions and morbid sensibility, I commenced the treatment with small doses of blue pill and ipecacuanha, from which no immediate benefit appeared to result. I then put him upon a month's course of nitrate of silver, with a view to allay the morbid sensibility of the mucous membrane.* The effect in this, as in almost every other case in which I have tried it, was surprizing and gratifying in the extreme. This patient, in common with many others who had taken this medicine, warmly expressed the great relief from irritation, flatulence, cutaneous chillness, discomfort, which it promptly procured them. This gentleman assured me that he had not felt himself so much in possession of the *sensation* of health, so far back as his memory could carry him, as he did since he began the use of the medicine in question. I have once or twice laid it aside from fears of its discolorizing property; but, after a while, returned to it, and always with augmented benefit. And this young man who, when he came to me, did not dare to go into company, partly from the depression of his spirits, partly from his well-founded terror of being led into dietetic excess, partly from the disastrous effects which the moral excitement of society ever had on his enfeebled and irritable nervous system; who, when I first saw him, was plunged into the deepest despondency and despair, is now cheerful and confident,—mingles freely in society, and looks and feels like one in excellent health." 202.

This is a very striking case, and we think we may pretty confidently say that the oxyde of silver will be found equally beneficial in gastric derangements, with little or no risk of discoloration of the skin.

---

* See Dr. J. Johnson's work on Morbid Sensibility, pages 43, 84, 85.

2. *The Dyspeptic's Melancholy Tale.*—"There is a class of patients who enter the apartments of physicians, with a mixed air of timorousness, reserve, peevishness and impatience. Their emaciation, if they are emaciated, is not of a ghastly character; something in their gait or countenance announces in general to the eye of the physician, that the malady of his visitor is not of an exceedingly urgent or of a violent nature. The fretfulness and restlessness engendered by the disease, hinders the patient from devoting much time to the civil ceremonial of introduction or explanation; but almost without a question on the physician's part, hurries him abruptly in *medias res.* It is easy to perceive, from the fluency and fulness with which the invalid pursues the history of his feelings and symptoms, that that history is rehearsed neither for the first nor fiftieth time. Sufferings usually stated as unprecedented; plans of diet suggested to him, and followed with various success; changes made from time to time in medicines and physicians; benefit derived from this doctor but not from that, from this watering place, but not from the other; total inability to enjoy society or life; gradual departure of the powers of intellectual application, growing hebetude of thought; a resolution of embarking in a totally new mode of life; of exchanging town for country, active and civic, for rural life; misery, and, by no means infrequently, a propensity to suicide,—these, or some of these, are the staple heads of a dyspeptic's inaugural discourse to any physician whom he may visit for the first time. The whole is delivered in a tone slightly querulous, the consequence of a physical and mental irritation of a sort entirely peculiar." 43.

A large class of these dyspeptics seem to experience as much pleasure in rehearsing their catalogue of miseries, as an infant does in sucking its thumb!

3. *Dyspeptic Phthisis.*—Our author appears to go far beyond Dr. Philip in respect to the influence of *disorders* of the stomach in inducing *diseases* of the lungs. We shall introduce one of the proofs adduced in support of the doctrine.

"—— ——, 22 years of age, and born of healthy parents; had suffered for several years from unpleasant sensations in the stomach; heat, pain, heaviness after meals. His tongue was clean; his bowels variable; easily deranged; his belly tense; his urine natural; his pulse 80, small; he was irritable, nervous, easily exhausted; easily chilled. His chest was perfectly normal. This fact I repeatedly ascertained, and that within ten days of his death. He had horrid dreams.

By treating him with sedatives and antispasmodics, and the mildest laxatives, the dyspeptic symptoms were greatly relieved; but he did not gather flesh, although his strength improved, and any departure from a very strict regimen immediately re-produced great gastric irritability and suffering. He constantly complained of chillness. On this account, I cautioned him against exposure to cold; aware of the truth of Andral's remark, that when the system has been long kept in a state of debility and febrility by a gastric irritation, any pulmonary affection is apt to be rapid and destructive.

In May, —— ——, was seized with influenza. A cavernous rhonchus was heard on the fourth day, and the fifth terminated his life.

*Inspection.*—The stomach, which had tortured ——'s existence for many years, was perfectly sound!! so was the liver; so were the intestines. The left lung was entirely disorganized; almost one purulent sac. The right lung was greatly diseased. And all this in the course of five days, not from a regular pneumonia or ungovernable pleurisy, but from ordinary influenza. It was clear that, in this case, the long constitutional irritation produced by the merely functional gastric affection, had by the maintenance of a constant febrility, annihilated the conservative vigour of the system. No doubt, had the brain been the organ of attack, structural change would have taken place in it as rapidly as it did in the lungs." 276.

We confess that this line of argument savours a good deal of the *post hoc ergo propter hoc*. Much confidence as we have in auscultation, we should be very sorry to pronounce lungs to be positively sound because we could detect no disease there. Have not lungs often broken down rapidly under inflammation, without any previous dyspepsia? Dr. D. says the *right* lung was "greatly diseased." If he had stated the nature of the diseased condition, we should have been better able to form an opinion as to that lung being perfectly sound five days before the death of the patient. Be this as it may, we quite approve the following direction.

"Our rule in all doubtful cases is, to use carefully the stethoscope; to consider carefully the history of the origin of the disease; to determine the absence or presence of pain or tumefaction in the abdomen; to watch if attention to the digestive organs, and the subjugation of irritation there, dissipate the pulmonary phenomena. We idle our time, if we treat a cough, sympathetic of gastric irritation, by measures directed exclusively to the chest. But, on the other hand, we may err fatally in mistaking for a merely secondary affection, an incipient and true phthisis, which sometimes signalises its commencement, by simultaneous gastric symptoms, whose more marked, but comparatively unimportant presence, may mask the graver malady." 281.

If Dr. Dick saw a tithe of those who, in the second or even third stage of phthisis, present themselves to us, labouring as they say, and as they have been told, under "STOMACH-COUGH," "but no affection of the lungs," he would be a little more guarded in his doctrines on this point. We will not say (though we might do so with a clear conscience) that this doctrine is the annual destruction of thousands in this country; but we may aver that it ruins the reputation of many young medical practitioners every year. Patients come to them with cough. Among the first questions is this. "Have you had any indigestion, or affection of your stomach, before the cough began?" "Oh, yes, Sir, I have indigestion for years before." With the "stomach cough" doctrine in his mind, the inexperienced practitioner naturally says to himself, here is a clear case. The indigestion was the cause,—the pulmonary disease is the effect. "Sublata causa, tollitur effectus," is philosophical, and that rule I will pursue. Meanwhile the patient dies of unequivocal phthisis, and as other medical men will probably have been consulted, the philosophical doctor —the *doctrinaire*—will be severely commented on, for telling the patient and friends, that it was merely a "stomach cough."

Now all we recommend is this:—If you find gastric and pulmonary disorder *co-existent*, do not be led away by the affirmations of the patient, that the *former* preceded the *latter*, and prescribe mutton chops thrice a day, with sherry and the shower-bath—not forgetting sulphate of iron and Epsom salts—but treat both complaints. The practitioner may depend upon it that the pulmonary affection is the main one, whether original or secondary, and that, whether he consults his patient's health or his own reputation, he will do well to direct his principal attention to the chest. Too many of the medical world is too apt to treat diseases accordin to the name which they affix to them in their own minds. If they determine that it is " INDIGESTION," then a certain routine diet and pharmacy are ordered which, in general, is the very reverse of that which is necessary in incipient pulmonary affection, where inflammatory action is present in nineteen cases out of twenty. The consequences need not be told! We do not accuse our author of these indiscriminations; but assuredly his writings will lead hundreds of inexperienced practitioners astray. We strongly recommend the following judicious advice to our younger brethren.

" As a general rule, it may be laid down, that so long as the secondary affections are *purely sympathetic, purely functional,* perseverance in the measures addressed to the digestive organs is alone requisite. This rule applies equally to the sympathetic affections of the heart, lungs, and brain. But it is incumbent on us to recollect, that it is almost inconceivable how insidiously sympathetic, erects itself into fixed and independent disease; which attention to the digestive organs may indeed palliate, but can then no longer eradicate." 302.

This is a good rule, but the one we have alluded to is better—namely, to treat both affections simultaneously. On this plan it is hardly possible to do harm.

We may conclude, with again expressing our opinion that Dr. Dick has, with great labour and no trifling discrimination, compiled, selected, compared, and, as it were, amalgamated, a vast store of " useful knowledge " on the subject of stomach-complaints, through which a large current of original matter constantly flows.

